# Multimodal Neuroimaging as a SUDEP Predictor: What Is Known and What Still Needs to Be Uncovered?

**DOI:** 10.1111/ene.70101

**Published:** 2025-04-12

**Authors:** Paolo Quintieri, Fedele Dono, Giacomo Evangelista, Clarissa Corniello, Sara Cipollone, Sibilla De Angelis, Antonio Ferretti, Stefano L. Sensi

**Affiliations:** ^1^ Department of Neuroscience, Imaging and Clinical Science “G. d'Annunzio” University of Chieti‐Pescara Chieti Italy; ^2^ Epilepsy Center, Neurology Institute “SS Annunziata” University Hospital University of Chieti‐Pescara Chieti Italy; ^3^ Behavioral Neurology and Molecular Neurology Units, Center for Advanced Studies and Technology (CAST) and Institute for Advanced Biomedical Technologies (ITAB) University of Chieti‐Pescara Chieti Italy

**Keywords:** connectivity, dysautonomia, metabolism, perfusion, SUDEP, volume

## Abstract

**Introduction:**

Sudden Unexpected Death in Epilepsy (SUDEP) is the most common cause of death in patients with poorly controlled epilepsy. To date, a higher risk of developing SUDEP is mainly identified by clinical factors, among which generalized tonic–clonic seizures and their frequency stand out as part of the highly debated SUDEP‐7 Scoring. This review investigates the role of neuroimaging‐based approaches as a tool to help predict SUDEP.

**Methods:**

We carried out a systematic search of the literature to identify multimodal neuroimaging modifications (i.e., MRI, fMRI, PET, and SPECT) in patients with epilepsy who died from SUDEP. The following databases were used: PubMed and Google Scholar. The review was registered on the PROSPERO platform (Registration code: CRD42024558765).

**Results:**

Fifteen articles were selected, investigating 104 SUDEP cases compared with 792 non‐SUDEP epileptic patients and 280 healthy controls (HC) (overall mean age 33.9 ± 1.6). Results suggest that SUDEP and non‐SUDEP cases and HC differ anatomically and functionally. In the SUDEP population, MRI data indicate relevant volume differences in the gray matter of the hippocampus and cerebellar cortices. In addition, functional imaging reveals discrepancies in network modulation within the brainstem and its relationship with several cortical structures. Although less consistent, PET and SPECT scan data point toward alterations in metabolism and perfusion in the frontal and brainstem areas.

**Conclusion:**

The reviewed data support correlations between the occurrence of SUDEP and neuroimaging multimodal alterations that could be relevant in death prediction.

## 1 Introduction

Sudden Unexpected Death in Epilepsy (SUDEP) is an occurrence leading to the sudden demise of healthy people with epilepsy (PwE). The condition occurs at a rate of up to 1.2 per 1000 PwE annually. The incidence of SUDEP in young adults (aged 20–45 years) is greater than sudden death in the general population by a factor of 27 [1]. Uncontrolled generalized tonic–clonic seizures (GTCS) are the most prominent risk factor for SUDEP [2] as well as living alone/sleeping alone [3].

The pathophysiological pathways contributing to the onset of SUDEP remain largely unknown. According to the literature, dysregulation of the autonomic nervous system (ANS), which leads to central hypoventilation and cardiac dysrhythmia, plays a pivotal role [4]. Thus, assessing ANS functioning in PwE is mandatory, particularly in the PwE subset with a high clinical risk for SUDEP.

Noninvasive assessments include blood pressure and heart rate variation measurement during postural changes, the Valsalva maneuver, sudomotor functioning testing, heart rate variability (HRV) evaluation, and neuroimaging techniques [5, 6]. Magnetic resonance imaging (MRI) studies indicate that PwE who died from SUDEP and patients with a high clinical risk thereof exhibit some structural changes in the brainstem and other regions of the Central Nervous System (CNS) involved in the Central Autonomic Network (CAN) [7]. Several functional and metabolic alterations have also been described with functional MRI (fMRI) [2] and Positron Emission Tomography (PET) [8]. Moreover, altered perfusion patterns with Single Photon Emission Computed Tomography (SPECT) have also been described [9]. However, SUDEP literature reveals controversial findings.

This review offers an overview of the potential structural, functional, metabolic, and perfusion alterations that PwE undergo before developing SUDEP.

## 2 Methods

The research was conducted per the PICOS criteria (Table S1).

The results of our systematic review were reported following the guidelines of the Preferred Reporting Items for Systematic Reviews and Meta‐Analyses (PRISMA) statement.

Two authors (P.Q. and G.E.) performed independent research from the latest available data until May 2024 using the following terms: (“SUDEP” OR “Sudden Unexpected Death In Epilepsy”) AND (“MRI” OR “Magnetic Resonance Imaging” OR “fMRI” OR “functional MRI” OR “PET‐CT” OR “Positron Emission Tomography‐CT” OR “SPECT” OR “Single‐photon emission computed tomography”).

The following electronic databases and data sources were systematically searched: MEDLINE (accessed through PubMed) and Google Scholar.

As per the inclusion criteria, we selected: (1) articles written in English, (2) studies that reported at least one neuroradiological imaging modality analysis (e.g., MRI, fMRI, SPECT, or PET‐CT), and (3) studies that reported neuroimaging analysis in SUDEP patients or those at high or low risk of SUDEP, possibly compared to healthy controls.

We performed individual and comprehensive quality assessments for each study, looking for selection biases (i.e., whether the diagnostic methods for SUDEP cases were described and which groups were analyzed) and measurement biases (i.e., on which basis the distinction between high‐risk and low‐risk patients was made).

The quality of the included studies in the meta‐analysis was assessed using the Newcastle‐Ottawa Quality Assessment Scale (NOS) [10], which ranges from 0 to 9. Studies that scored ≥ 4 were considered to be of good quality. Nine reviewers (P.Q., F.D., G.E., C.C., S.C., S.D., D.L.) independently reviewed the retrieved articles. Disagreements were discussed and resolved collegially.

We extracted and collected the following patient data: age, sex, and risk of epilepsy worsening into SUDEP according to the SUDEP‐7 Inventory [10].

SUDEP classification (definite, possible, probable, none), SUDEP score, seizure semiology, seizure frequency, seizure etiology, epilepsy syndrome diagnosis, EEG features, ASM therapy (number, type, and dosage), treatment duration, surgery of epilepsy (Yes or No), Vagal Nerve Stimulation (Yes or No), MRI, fMRI, PET, and SPECT features.

The final study protocol was registered in the International Prospective Registry of systematic reviews PROSPERO (Registration code: CRD42024558765).

### 2.1 Statistics

Statistical analysis was performed on the final data set containing all individual patient data extracted from the included studies. We conducted a pooled analysis using descriptive statistics (mean ± SD, frequency) to represent the pooled data set. The neuroradiological findings of the different groups (SUDEP, High and Low‐Risk, healthy controls) were compared.

## 3 Results

### 3.1 Literature Search

**FIGURE 1:**
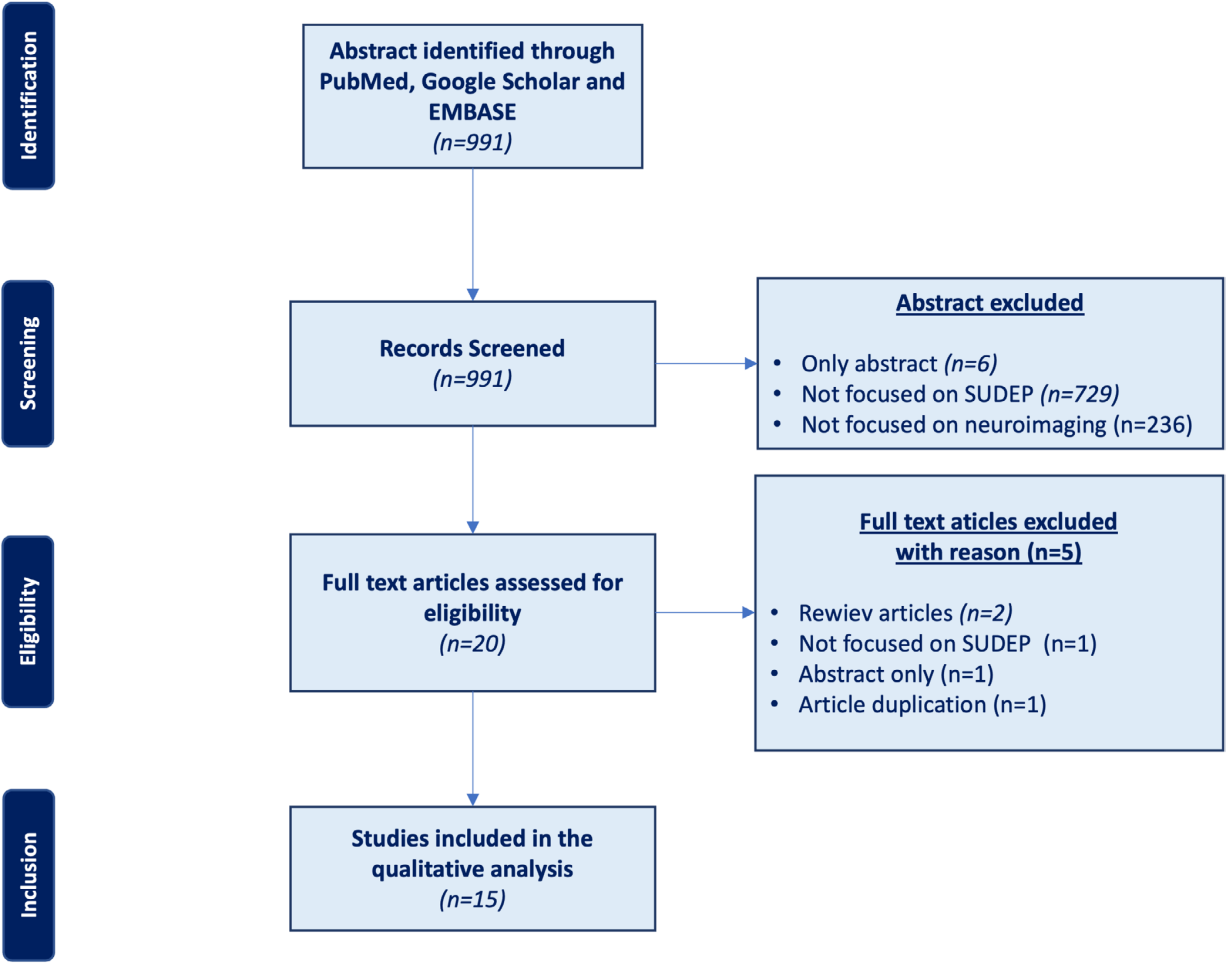
Preferred Reporting Items for Systematic Reviews and Meta‐Analyses diagram (PRISMA). From the 991 articles screened, 15 articles were finally included in the literature review. SUDEP: sudden unexpected death in epilepsy.

The literature search yielded 991 articles (MEDLINE: 115 results; Google Scholar: 876). After the screening, the full texts of 20 articles were reviewed for eligibility. Of these, 5 articles were excluded (see Figure 1 for reasons for exclusion). Only 15 reports (i.e., 15 case–control studies) [2, 7, 8, 9, 11, 12, 13, 14, 15, 16, 17, 18, 19, 20, 21] met the selection criteria and were considered eligible for the review (Figure 1).

The quality assessment revealed that the risk of selection bias was reasonably low, as almost all studies used age‐ and gender‐matched healthy controls, and all patients were diagnosed with epilepsy according to the ILAE criteria. Likewise, the ILAE criteria were applied to define SUDEP cases [22]. On the other hand, not all studies distinguished between high‐and low‐risk cases of SUDEP according to a definite SUDEP‐7 scoring when applied to studied patients.

According to the Newcastle‐Ottawa Quality Rating Scale (NOS), 8 reviewed papers were assigned a score of 6, 2 papers were assigned a score of 5, and 5 papers were assigned a score of 4 (detailed scoring results are given in Table S2).

Details of the included papers are reported in Figure 1.

### 3.2 Clinical and Demographic Characteristics

Throughout the reviewed articles, 1176 subjects were considered and classified according to their status as definite SUDEP cases (*n* = 104, 71 males), non‐SUDEP epileptic patients (*n* = 792, 414 males), and healthy controls (*n* = 280, 92 males). Most studies evaluated the frequency of GTCS (> 3 per year), duration of epilepsy (> 15 years), and age at onset (< 16 years of age) to separate high‐and low‐risk subjects without providing a definite cutoff score. Two articles applied the same criteria to the studied cohort. They generated an Odds Ratio (OR) score for each patient, identifying high‐and low‐risk subjects according to an OR cutoff value of 3.9. Only two reviewed studies stratified living patients based on a SUDEP‐7 Inventory cutoff score, amounting to 5 in both cases.

Overall, in terms of seizure manifestation, about one third of the patients who had succumbed to SUDEP experienced GTCS or focal to bilateral tonic–clonic seizures, with the remnant epileptic manifestations being classified as having either a focal or unknown origin (Table 1).

**TABLE 1:** Demographics and clinical data.

	SUDEP cases	Non SUDEP epileptic patients	Healthy controls
	Total patients (*n* = 104)	Total patients (*n* = 792)	Total patients (*n* = 280)
Age (years)	31.7 ± 6.9	35.7 ± 3.1	34.5 ± 2.2
Sex (M)	71	414	92
Etiology			
Unknown etiology	39	617	/
Known etiology	65	175	/
Structural	23	124	/
Type of seizure			
Only Focal	50	529	/
Focal plus Focal‐to‐bilateral	9	130	/
Generalised Tonic–clonic	19	123	/
Non available	26	10	/
Epilepsy type			
TLE	33	260	/
FLE	14	72	/
IGE	9	19	/
Not available	48	441	/

Abbreviations: FLE, frontal lobe epilepsy; IGE, idiopathic generalized epilepsy; TLE, temporal lobe epilepsy.

Single data analysis was not available to determine a clear‐cut separation of patients at risk of developing SUDEP according to seizure semiology, recurrence, and etiology.

### 3.3 Imaging Studies Methods

#### 3.3.1 MRI

Of the eight studies that explored anatomic organizational differences between the assessed groups, six employed a 3 T MRI [7, 12, 14, 19, 20, 21], one used a 4 T MRI [13], and the results of the last one were obtained through a 9.4 T MRI [11]. All but one of the articles [20] omitted to report information regarding the time span between MRI recording and SUDEP occurrence. Patients and controls were assessed with high‐resolution T1 scans to study brain volumes and structural alterations. One study focusing on temporal lobe epilepsy associated with mesial temporal sclerosis (TLE‐MTS) based its imaging on T2‐weighted high‐resolution images to confirm or disprove the hypothesis of hippocampal sclerosis. Most subjects who were studied with 3 T or 4 T MRI underwent fast spoiled gradient‐echo (FSPGR) 3D‐T1 scans (repetition time = 8.3, echo time = 3.1, slices = 170, slice thickness = 1.1 mm, matrix size = 256 × 256, field‐of‐view = 240 × 240 mm) with 20 min as the most frequent total acquisition time. Structural analyses were conducted using Voxel‐based morphometry (VBM) through Statistical Parametric Mapping (SPM12). Images were normalized to the MNI template space and segmented into white matter, gray matter, and CSF tissue classes. One study [21] employed standard MRI for detecting broad structural changes of the amygdala and diffusion MRI to provide a detailed account of microstructural modifications. Based on the notion that axonal organization directs water diffusion, the technique employs parameters such as orientation dispersion index (ODI) and neurite density index (NDI) to define the orientation of axons and determine the organization of white matter bundles. To achieve this, diffusion metrics were obtained by fitting neurite orientation dispersion and density imaging (NODDI) models. Another study [20] explored the correspondence between long‐lasting epileptic activity and dysautonomia using Heart Rate Variability (HRV) as an indicator of SUDEP probability. Deformation‐based morphometry of the brainstem was applied to MRI scans obtained from epileptic patients, controls, and SUDEP patients, employing the following parameters: TR/TE/TI = 2300/2.96/1000 ms, 1.0 × 1.0 × 1.0 mm^3^, acquisition time 5.30 min. A profile similarity approach (PSI approach) was subsequently applied to describe the pattern of volume loss in patients who died from SUDEP, and results were compared to those obtained from the other groups. All subjects underwent simultaneous ECG recording during their imaging exam.

Finally, the study conducted with a 9.4 T scanner [11] carried out postmortem analyses on whole or hemi‐brainstem blocks, obtaining T2‐weighted images via a multi‐slice spin echo (SEMS) with an adopted echo time/repetition time (TE/TR) of 35/8000 ms. A multi‐TR saturation recovery approach was applied using SEMS (TE = 12 ms and TRs = 0.35/0.5/0.85/1.5/3/9.5 s) to perform T1 mapping. The scanning protocol, including the adjustments, took 39 h. The brainstem MRI scans were followed by the axial cutting of the medulla in serial sections with 20 μm thickness using the Tissue‐Tek AutoSection automated microtome and their subsequent staining with cresyl‐violet (CV) to implement volume assessment of five specific regions entailed in respiratory control. The volume of these regions, identified as Regions of Interest (ROIs), was evaluated with the application of the Cavalieri method (StereoInvestigation software, MBF Biosciences) on sections selected for volume estimation, ultimately carried out by aligning the MRI image slices with the corresponding sections. When whole brainstem blocks were analyzed, volume measurement was conducted on either side. The five regions undergoing the ex vivo analysis were the Reticular formation Zone (RtZ), the Ventrolateral Medulla (VLM), the Medullary Raphe (MR), the Inferior Olive (IO), and the Solitary Tract (ST).

#### 3.3.2 fMRI

Brain functional connectivity was investigated using fMRI resting‐state scans based on the blood oxygen level‐dependent (BOLD) technique [2, 15, 16, 17]. No information was reported regarding the time span between fMRI recording and SUDEP occurrence. All scans were obtained through a 3 Tesla machine and accompanied by EEG recordings spanning 10 or 20 min. No specific tasks were performed during the fMRI acquisition. All patients underwent resting‐state fMRI for at least 10 min. The fMRI scans were mostly performed through a gradient‐echo echo‐planar‐imaging sequence with the following characteristics: repetition time (TR) = 3000 ms, echo time (TE) = 30 ms, flip angle = 90°, matrix size = 64 × 64, field of view (FOV) = 24 × 24 cm, slice thickness = 3 mm, 44 slices, voxel size = 3 mm × 3 mm × 3 mm. Two studies adopted slightly different acquisition parameters (i.e., TR = 2000 ms, TE = 30 ms, flip angle = 90°, matrix size = 64 × 64, FOV = 24 × 24 cm, slice thickness = 5 mm, 30 slices in one case; TR = 3000 ms, TE = 30 ms, flip angle = 90°, matrix size = 64 × 64, FOV = 24 × 24 cm, slice thickness = 2.4 mm, 44 slices, voxel size 3.75 × 3.75 × 3 mm). EEG was performed in all studies except for one. Of the three studies employing EEG, two used a 64 channels cap, while the remaining one used a 32 channels cap. A seed‐based functional connectivity (FC) analysis was carried out, with seeds consisting of regions with a relevant role in autonomic and respiratory regulation (i.e., anterior cingulate cortex, anterior insula, posterior insula, thalamus, amygdala, hippocampus, parahippocampal gyrus, precuneus/posterior cingulate cortex (PCu/PCC), cuneus, caudate, putamen and Brodmann area 25). In two cases, the assessment of these regions was based on parcels (i.e., nonoverlapping contiguous regions) from the Brainnetome atlas. One study employed the Harvard‐Oxford (HO) cortical and subcortical atlas. In one study, further analyses were conducted, including network modularity, nodal participation, nodal degree centrality, hub prevalence, and hub distribution index.

#### 3.3.3 PET

The two studies [8, 18] concerning metabolic alterations in SUDEP and epileptic patients involved a group of high‐and low‐risk patients, as well as those who died from SUDEP. These patients underwent interictal 18‐fluorodeoxyglucose (FDG) PET as part of their presurgical evaluation. No information was reported regarding the time span between PET scan acquisition and SUDEP occurrence. PET scans were performed after at least 6 h of fasting. EEG was performed using surface electrodes during tracer injection and the entire FDG uptake period. The FDG injection lasted about 40 min in all cases and was followed by 20 min of scan, with brain data acquired in 3D mode. In all cases, a nonlinear normalization of the acquired PET scans was conducted to fit a template in the ICBM/MNI space. The process employed image modulation to correct possible nonlinear spatial warping during the normalization. Afterward, normalized images were resampled at 3 mm isotropic resolution.

#### 3.3.4 SPECT

Perfusion patterns were the object of only one study in our review [9].

In subjects at risk of developing SUDEP, a tendency toward hypoperfusion or hyperperfusion of specific brain regions was enquired both in the ictal and the interictal phase, thanks to the quantification of cerebral blood flow in multiple cortical and subcortical areas, later grouped into two major Regions of Interest (ROIs): the hyperperfused and the hypoperfused areas. Interictal SPECT scans were obtained after an interval of at least 24 h from the latest seizure. In contrast, video‐EEG monitoring allowed the detection of the onset of seizures and consequently produced ictal imaging within 40 min from the electroencephalographic findings.

### 3.4 Imaging Results

#### 3.4.1 MRI Findings

Eight articles concerning MRI findings were reviewed [7, 11, 12, 13, 14, 19, 20, 21].

As for strictly structural differences between the studied categories, cerebellar volume loss was consistently reported in SUDEP cases, both in vermal and neocerebellar portions. On the other hand, the MRI of high‐risk subjects indicated that a similar atrophic process had already started but had not yet fully completed, given the relatively spared cerebellar neocortex contrasting with the already shrunk vermis [7]. It should be noticed, however, that these high‐risk patients had undergone a more abundant previous anti‐seizure therapy, making it unclear the extent to which the imaging changes were the product of epilepsy alone. A similar finding that would prove the apparent time‐related graduality in anatomical alterations is the volume reduction of thalamic pulvinar and posterior hippocampal gray matter present in definite SUDEP patients but absent in high‐risk patients [13].

Aside from significant shrinkage of certain encephalic areas, another pathological variation presented as a significant SUDEP indicator is volume gain, as is the case with the bilateral amygdala and entorhinal cortex, which tend to show an increase in gray matter volume in the SUDEP and high‐risk classes. Interestingly, diffusion studies pointed to a preferential left amygdala microstructural disarray, as highlighted by a reduction in the packing density of neurites and their spatial orientation (NDI and ODI, respectively) in epileptic patients compared to controls [21]. Similarly, despite its tendency for global structural decline, several authors agree on describing the hippocampus and the parahippocampal gyrus as swollen structures, both in definite SUDEP cases and in patients exposed to a higher probability of SUDEP [14, 19].

The brainstem is often reported as a suspected major actor in SUDEP development, as its volume depletion may account for the failure of those systems implied in autonomic control and cardiac arrhythmias. Results would implicate that TLE, particularly when associated with mesial temporal sclerosis (TLE‐MTS), is the leading cause of mesencephalon volume loss and brainstem functional impairment [13].

Evidence of clinical significance of brainstem imaging abnormalities comes from reduced HRV values observed in epileptic patients who showed various degrees of brainstem volume loss, mainly localized in the periaqueductal gray (PAG) and autonomic nuclei (i.e., nucleus ambiguous, nucleus of the solitary tract, dorsal motor nucleus of vagus nerve, hypoglossus, parabrachialis, caudal portion of reticular formation). Of further interest, SUDEP patients scans highlighted a certain graduality of brainstem volume loss, tracing an inverse relation between the level of atrophy and the time to SUDEP, suggesting the possibility of predicting progression to SUDEP according to the amount of atrophy detected [20].

**FIGURE 2:**
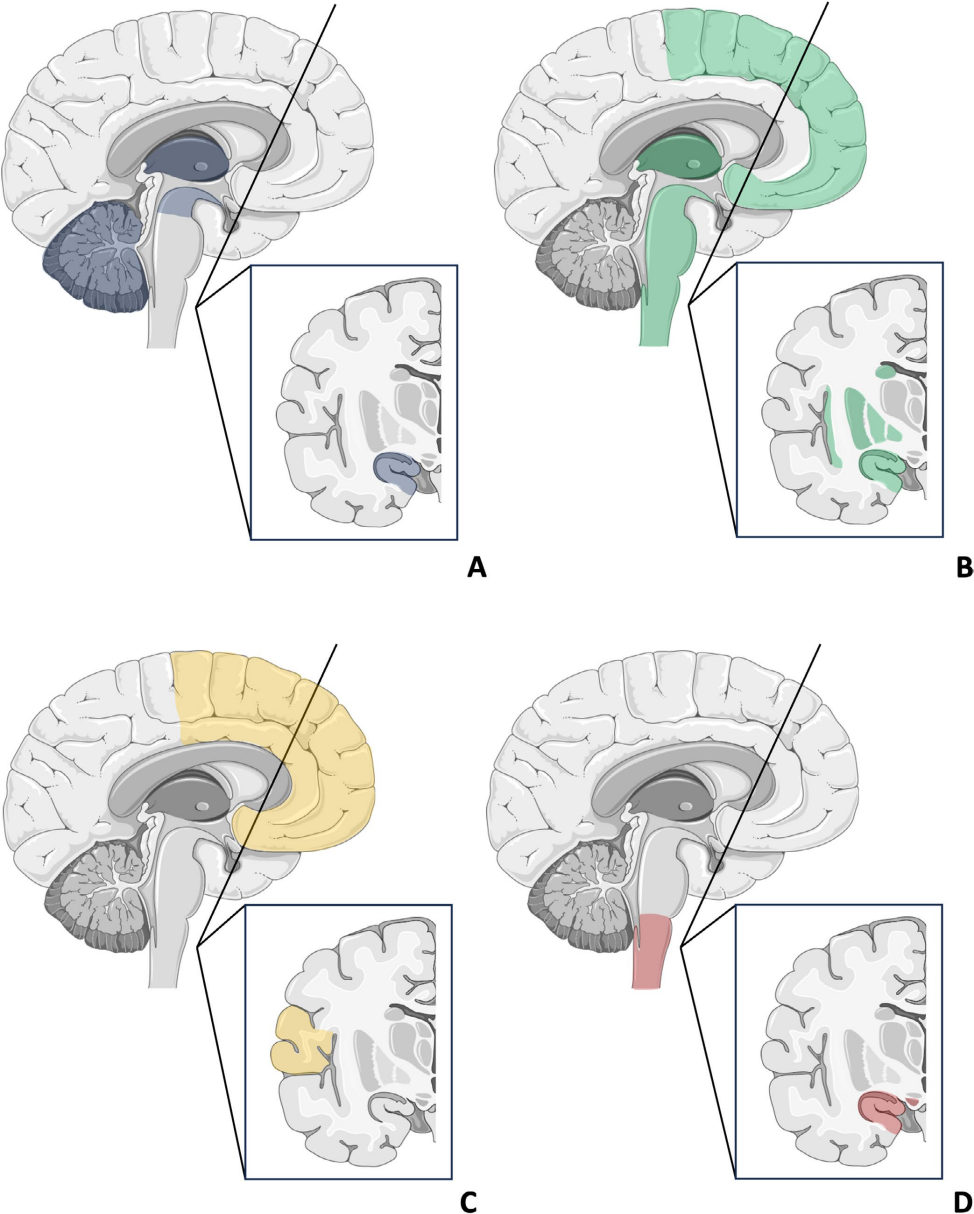
Multimodal neuroimaging assessment of patients who died from SUDEP. (A) MRI changes; (B) fMRI changes; (C) PET changes; (D) Interictal‐Ictal SPECT changes. See the text for the description. (SMART‐Servier Medical ART was used to create the artwork).

Graphical representation of MRI structural changes in SUDEP patients is depicted in Figure 2A.

#### 3.4.2 fMRI Findings

The link between structural loss and functional alterations is better explained when considering the fMRI results of the four examined studies which assessed brain network disruption [2, 15, 16, 17]. One such network is the brainstem regulatory subnetwork whose modularity (i.e., how a specific network is organized into separate modules) exhibited a deficit in SUDEP cases and high risk for SUDEP PwE [15]. The studies stressed the tendency of deep structures, more prominently the thalami [2], to increase nodal participation, a parameter accounting for the degree of intermodular connection and, thus, a marker of the involvement of a single region outside the module it belongs to. Degree centrality, a measure of the arrangement of brain networks around specific hubs, displayed characteristic patterns of abnormality in SUDEP cases and high‐risk subjects in contrast with low‐risk and healthy subjects. Functional imaging in the former groups revealed a reduced centrality degree in the insular and cingulate cortices and an opposite pattern in the hippocampus [2]. As for the total number of hubs and their functional coordination in the brainstem subnetwork, hub prevalence and hub distribution indices showed greater levels in low‐risk subjects, possibly relating to compensative mechanisms counteracting the described functional decline [2]. Another extensively studied fMRI feature potentially reflecting pathological changes occurring in a seizure‐exposed brain is Functional Connectivity (FC). FC indicates the coactivation levels of different brain areas at resting state or during the execution of specific tasks. Various subnetworks exhibited a modified functional connectivity pattern in high risk for SUDEP and SUDEP PwE. Increased FC was observed in the subcortical nuclei, the medial prefrontal cortex, the hippocampus, and the insula, whereas the brainstem and the amygdala showed opposite tendencies [2]. Other altered networks included those coordinated by the thalami, whose connection with brainstem structures, especially with the pons and the midbrain [16], was considerably reduced.

Graphical representation of fMRI connectivity changes in SUDEP patients is depicted in Figure 2B.

#### 3.4.3 PET Findings

The effect of repeated bilateral tonic–clonic seizures on cerebral metabolism is of great interest. Two reviewed articles [8, 18] indicated that high‐risk PwE exhibited hypometabolism in three fundamental clusters, detected in the medial right and left inferior frontal cortices. PET scans conducted on a small number of SUDEP PwE also revealed bilateral frontal hypometabolism. Conversely, specific alterations were not found in low‐risk cases. Another study evaluated seizure recurrence and pointed out an inverse correlation between the bilateral 18‐FDG uptake in frontal areas and the frequency of tonic–clonic seizures [8].

Graphical representation of PET changes in SUDEP patients is depicted in Figure 2C.

#### 3.4.4 SPECT Findings

Only one study investigated SPECT analyses in PwE, establishing a comparison between High and Low risk for SUDEP cases [9]. In the paper, twenty‐nine analyzed interictal‐ictal SPECT scans came from patients with drug‐resistant focal epilepsy. The scan results were interpreted within a perfusion continuum, ranging from hypoperfusion to hyperperfusion.

The ictal to interictal phase should be regarded as a critical moment in generating a vascular upheaval, with cerebral areas like the red nucleus, the substantia nigra, the medulla, and the entorhinal area potentially undergoing significant reductions in perfusion. Interestingly, most of the same hypo‐perfused structures were functionally altered on fMRI scans.

Graphical representation of SPECT changes in SUDEP patients is depicted in Figure 2D.

## 4 Discussion

The aim of assessing cerebral anatomical and functional modifications in patients who died from SUDEP is to understand its pathophysiological causative processes.

Several brain structures are distinctly involved in SUDEP; the brainstem, the thalami, and the cerebellum are among the mainly reported ones. These structures are deeply interconnected and are pivotal in breathing and cardiovascular functioning. Preclinical studies have shown that the cerebellum modulates blood pressure changes, favoring recovery from apnea and hypercarbia through the stimulation of diaphragm contraction and airway opening [7]. Thus, the impairment of these functions due to cerebellar atrophy may represent a major risk factor for SUDEP. In SUDEP animal models, a constant state of neuronal over‐excitability could result in hyperactivation of Purkinje cells from pontine long climbing fibers, leading to neurotoxicity and, consequently, the inability to prevent respiratory failure. Similarly to Purkinje cells, left hippocampal CA1 damage induced by prolonged seizures has been demonstrated to lead to heart rate and breathing abnormalities [7].

Other regions undergoing atrophy are also involved in cardiorespiratory alterations. For instance, the left posterior thalamus modulates the response to low oxygen levels and hypercapnia. Together with the ventrolateral Periaqueductal Gray (PAG), the thalamus participates in breathing control through timed signals that couple cerebellar activity with respiratory input, showing a hierarchical rostrocaudal organization of breathing recovery centers [13]. Repetitive exposure to seizures amplifies apnea‐inducing pathways. Such would be the case with the amygdala, a hypertrophic region in TLE‐MTS patients that induces hypotension and plays a further precipitating role in patients with an inefficient cerebellar system [14].

Most of the areas described as anatomically altered seem to exhibit functional impairments, as analyzed through the assessment of fMRI parameters. The fMRI findings indicate decreased modularity in the brainstem, suggesting a diminished capability to tackle sudden cardiac arrhythmias and apnea. The increased nodal participation of multiple thalamic regions supports the likelihood of seizure spreading [7]. It has been hypothesized that diverging values across the studied groups could underlie an inherent capability of some networks to dampen functional impairments. In low‐risk subjects, a rise in the number of brainstem hubs and their distribution indices was reported, a finding supporting a partial reorganizing and compensatory effort. An attempt at reorganization was also present in SUDEP and high‐risk patients, although less pronounced changes were appreciated [2]. Highly frequent seizures have also been connected with decreased FC between the thalami and the brainstem, hinting that a higher control of the brainstem driven by the oxygen‐sensitive thalamic nuclei is no longer capable of sending out the appropriate stimuli in patients risking SUDEP. Similarly, the thalamus‐anterior cingulate cortex (ACC) circuitry, necessary for blood pressure adjustment, has been found to hit sub‐functional activity levels due to repeated seizures [16], in a similar fashion to what was observed with regard to the ACC‐anterior insula circuitry [17]. The amygdala, indicated by different studies as a sensor of low oxygen levels and a promoter of inspiratory functions, showed a similarly depressed FC relation with the brainstem. On the contrary, the medial prefrontal cortex, which entertains blood pressure‐related connections with the insula and the hippocampus, showed increased FC values. Since this was primarily observed as resulting in HRV reduction and tachycardia, it is believed that its ultimate effect is the prevalence of sympathetic tone [15].

The shortage of studies concerning the use of PET and SPECT techniques in epileptic patients, especially in comparison with MRI and fMRI, impedes a complete understanding of metabolic and perfusion changes. The available studies have described decreased glucose metabolism in medial frontal regions, including the anterior cingulate gyrus. In the case of focal‐to‐bilateral seizures, such alterations seem to develop independently from the epileptogenic focus, since frontal hypometabolism is observed even when extra‐frontal epileptogenic foci are recognized. Evidence for that is the reported reversal of hypometabolism in the interested regions following temporal lobe surgery, which potentially prevents seizure generalization and spread to the frontal and cingulate cortices [18]. Other relatively less consistent metabolic findings regard increased FDG uptake in the basal ganglia, thalamus, brainstem, and cerebellum, although whether these changes are the result of repeated epileptic activity or a form of compensation remains unclear [8].

The perfusion patterns observed in the study employing SPECT mainly concerned the red nuclei, the substantia nigra, and the medulla. In PwE with recurrent focal‐to‐bilateral tonic–clonic seizures, these structures displayed a tendency to hypoperfusion both in the ictal and the interictal phases, presumably as a direct effect of seizures or as a reaction to long‐lasting epileptic activity [9]. Although not all the 29 scanned patients manifested the complete assortment of perfusion alterations, it may be relevant to point out that when present, perfusion abnormalities in different patients appeared to be equally divergent from nonpathological patterns (Table 2).

**TABLE 2:** Papers included in the systematic review and their main findings.

Paper	Methodology	SUDEP patients	Non‐SUDEP patients	Healthy controls	Findings in SUDEP and high‐risk
Patodia et al.	MRI	18	11	18	Loss of medullary volume
Allen et al.	MRI	25	48	25	Volume loss in cerebellum, hippocampus, left thalamus and posterior cingulate
Liu et al.	MRI	0	21	0	Postictal hypoperfusion
Mueller et al.	MRI	2	30	17	Volume loss in PAG and mesencephalic reticular formation
Wandschneider et al.	MRI	12	53	15	Increased amygdalo‐hippocampal gray matter volume
Neuhaus et al.	MRI	0	100	100	Bilateral amygdala and parahippocampal gyrus volume gain, cerebellar volume loss in epileptic patients.
Mueller et al.	MRI	26	18	11	Autonomic nuclei volume loss‐dependent HRV decrease in SUDEP patients
Legouhy et al.	MRI	0	143	53	Increased amygdala volume, left amygdala microstructural disarray as evidenced through diffusion‐weighted MRI sequences
Allen et al.	fMRI	8	32	16	Increased thalamic participation, altered brainstem connectivity and modularity
Allen et al.	fMRI	0	32	0	Decreased connectivity between thalami and amygdala, brainstem and ACC
Tang et al.	fMRI	0	25	0	Reduced thalamus‐midbrain and thalamus pons connectivity
Kassinopoulos et al.	fMRI	9	89	25	Reduced anterior insula‐ACC connectivity
Kumar et al.	PET	4	56	0	Bilateral frontal lobe hypometabolism
Whatley et al.	PET	0	125	0	Increased FDG uptake in basal ganglia, midbrain and pons
Chacon et al.	SPECT	0	9	0	Red nuclei and substantia nigra hypoperfusion, putamen hyperperfusion during the interictal and ictal state

## 5 Limitations

Our review was burdened by several limitations.

First of all, the included studies seldom reported actual information regarding seizure type. In fact, it is relevant to acknowledge the possibility that seizure foci yield abnormal results per se, at least in terms of perfusion and metabolism, and thus might not result from an actual dysfunction in the CAN structures. Moreover, it should be highlighted that SUDEP and high‐risk cases, due to their tendency to drug resistance, are usually treated with multiple ASMs. Atrophy, especially in the cerebellar vermis, can be at least partially explained by long‐course therapies (e.g., sodium channel blockers), making it unclear the extent to which the imaging changes are the product of epilepsy alone. Only one article described the pharmacological history of the studied PwE and indicated stronger ASM regimens in patients with the most relevant vermian atrophy. At the same time, it is unknown whether these drug regimens can influence functional parameters or their effects are merely limited to anatomy.

Psychiatric and behavioral disorders, including psychosis and depression, present with a certain recurrency in epileptic patients, and their possible influence over CNS functional parameters should be taken into account when assessing fMRI results. A review evaluating network dysfunctions in major depressive disorder (MDD) reports consistent frontoparietal hypoconnectivity across the studied patients, coupled with an increase of FC in the Default Mode Network (DMN). The latter is a large‐scale brain network encompassing several regions, most prominently the Posterior Cingulate Cortex (PCC), the prefrontal cortex, and the hippocampi [23]. The studies regarding fMRI scans did not specify whether epileptic patients were experiencing behavioral imbalances, nor was it determined if such patients had been under psychiatric drugs of any sort.

As for the methodology of the performed neuroimaging exams, only one study [20] reported how long before the patients' demise they were executed. Finally, patients were classified as high‐risk for SUDEP in all the reviewed studies, but one [20] lacked a practical evaluation of autonomic parameters, thus failing to provide a causality between neuroradiological findings and the actual clinical imbalance of the ANS.

## 6 Conclusions

Our review offers deeper insight into the pathological variations of brain structure, function, metabolism, and perfusion associated with SUDEP. Although radiological features are not currently included in the criteria for defining SUDEP or identifying high‐risk patients, the described imaging alterations over regions exerting cardiorespiratory control should be further investigated as potential markers of chronic epileptic activity.

## Author Contributions

**Paolo Quintieri:** investigation, writing – original draft, writing – review and editing, visualization. **Fedele Dono:** conceptualization, investigation, writing – original draft, methodology, validation, visualization, writing – review and editing, formal analysis, project administration, data curation, supervision. **Giacomo Evangelista:** methodology, writing – review and editing, data curation, supervision. **Clarissa Corniello:** writing – review and editing, investigation. **Sara Cipollone:** investigation, writing – review and editing. **Sibilla De Angelis:** writing – review and editing. **Antonio Ferretti:** writing – review and editing, formal analysis, project administration, data curation, supervision. **Stefano L. Sensi:** data curation, supervision, resources, funding acquisition, writing – review and editing, methodology, validation, visualization.

## Ethics Statement

We confirm that we have read the Journal's position on issues involved in ethical publication and affirm that this report is consistent with those guidelines.

## Conflicts of Interest

The authors declare no conflicts of interest.

## Supporting information


Table S1.

Table S2.

## Data Availability

The data that support the findings of this study are available from the corresponding author upon request.
