# Supplementary material for: Multimodal Neuroimaging as a SUDEP Predictor: What Is Known and What Still Needs to Be Uncovered?
Source: Eur J Neurol. 2025 Apr 12;32(4):e70101. doi: 10.1111/ene.70101 (PMC11992477; doi:10.1111/ene.70101)
Supplement: Supplementary file 1 — Table S1. [file ENE-32-e70101-s002.docx]

**P** People with epilepsy (PwE) who died for SUDEP. We considered the presence of comorbidity, SUDEP classification (definite, possible, probable, none), SUDEP score, seizure semiology, seizure frequency, seizure etiology, epilepsy syndrome diagnosis, neuroimaging (MRI, fMRI, PET, SPECT) features.

**I**  PwE who underwent neuroimaging (MRI, fMRI, PET, SPECT).

**C** PwE who did not die for SUDEP and healthy controls.

**O** Neuroimaging changing in patients died for SUDEP.

**S**  Systematic review and meta-analysis

**Supp. Tab. 1** PICOS criteria (Population; Intervention; Comparison; Outcome; Study design)
