# Supplementary material for: Multimodal Neuroimaging as a SUDEP Predictor: What Is Known and What Still Needs to Be Uncovered?
Source: Eur J Neurol. 2025 Apr 12;32(4):e70101. doi: 10.1111/ene.70101 (PMC11992477; doi:10.1111/ene.70101)
Supplement: Supplementary file 2 — Table S2. [file ENE-32-e70101-s001.docx]

| **Reference** | **Representativeness of the exposed cohort** | **Selection of the non exposed cohort** | **Ascertainment of exposure** | **Demonstration that outcome of interest was not present at start of study** | **Comparability of the cohorts included** | **Assessment of outcome** | **Was follow-up long enough for outcomes to occur?** | **Adequacy of follow up of cohorts** | **Total score** |
| --- | --- | --- | --- | --- | --- | --- | --- | --- | --- |
| *Patodia S. et al.,*  *2021* | * | * | * | * | * | * | - | - | ****** |
| Allen LA et al.,  2019 | * | * | * | * | * | * | - | - | ****** |
| Liu J et al.,  2020 | * | - | * | * | - | * | - | - | **** |
| Mueller SG et al.,  2014 | * | * | * | * | - | * | - | - | ***** |
| B Wandschneider et al.,  2015 | * | * | * | * | * | * | - | - | ****** |
| Allen LA et al.,  2019 | * | * | * | * | * | * | - | - | ****** |
| Allen LA et al.,  2017 | * | - | * | * | - | * | - | - | **** |
| Tang Y et al.,  2014 | * | - | * | * | - | * | - | - | **** |
| Kassinopoulos M et al.,  2023 | * | * | * | * | * | * | - | - | ****** |
| Kumar A et al.,  2021 | * | * | * | * | - | * | - | - | ***** |
| Whatley BP et al., 2021 | * | - | * | * | - | * | - | - | **** |
| Chacon LM et al., 2022 | * | - | * | * | - | * | - | - | **** |
| Neuhaus et al.,  2024 | * | * | * | * | * | * | - | - | ****** |
| Mueller et al.,  2018 | * | * | * | * | * | * | - | - | ****** |
| Legouhy et al,  2023 | * | * | * | * | * | * | - | - | ****** |

**Supp.Tab.2** Newcastle-Ottawa Quality Assessment Scale single paper score.
